# Predictors of Comorbid Conditions in Women Who Carry an *FMR1* Premutation

**DOI:** 10.3389/fpsyt.2021.715922

**Published:** 2021-10-01

**Authors:** Emily Graves Allen, Krista Charen, Heather S. Hipp, Lisa Shubeck, Ashima Amin, Weiya He, Jessica Ezzell Hunter, Katharine E. Shelly, Stephanie L. Sherman

**Affiliations:** ^1^Department of Human Genetics, Emory University School of Medicine, Atlanta, GA, United States; ^2^Department of Gynecology and Obstetrics, Emory University School of Medicine, Atlanta, GA, United States; ^3^Genomics, Ethics, and Translational Research Program, RTI International, Triangle Park, NC, United States

**Keywords:** FMR1, premutation, FXPOI, FXTAS, fragile X syndrome

## Abstract

**Purpose:** Women who carry an *FMR1* premutation (PM) can experience two well-established PM-associated disorders: fragile X-associated primary ovarian insufficiency (FXPOI, affects ~20–30% carriers) and fragile X-associated tremor-ataxia syndrome (FXTAS, affects ~6–15% carriers); however, emerging evidence indicates that some of these women experience complex health profiles beyond FXPOI and FXTAS.

**Methods:** In an effort to better understand predictors for these comorbid conditions, we collected self-reported medical histories on 413 women who carry an *FMR1* PM.

**Results:** There were 22 health conditions reported by at least 9% of women. In an exploratory analysis, 12 variables were tested in logistic regression models for each comorbid condition, including demographic variables, environmental variables, PM-associated factors, and endorsement of depression and/or anxiety. More than half of the comorbid conditions studied were associated with women who self-reported having anxiety. Age, smoking, body mass index (BMI), and depression were also significant predictor variables for specific comorbid conditions.

**Conclusions:** Age, smoking, and BMI were significantly associated with a subset of the comorbid conditions analyzed. Importantly, depression or anxiety were also significantly associated with many of the comorbid health conditions. This work highlights some of the modifiable factors associated with complex health profiles among women with an *FMR1* PM.

## Introduction

Individuals that carry an *FMR1* premutation (PM) allele (55–199 CGG repeats) are at risk for varied health consequences, some of which are unique to women. First, women who carry a PM, but not men, can be transmit an expanded full mutation (FM, >200 methylated repeats) to their offspring, leading to fragile X syndrome (FXS) (1). Fragile X syndrome is the most common genetic form of intellectual and developmental disability (IDD) and of autism spectrum disorder (2). Women with a PM are also at risk for Fragile X Premutation Associated Conditions (FXPAC) which include fragile X-associated primary ovarian insufficiency (FXPOI), fragile X-associated tremor-ataxia syndrome (FXTAS), and Fragile X-Associated Neuropsychiatric Disorders (FXAND) (3, 4). FXPOI, characterized as irregular or absent menstrual cycles due to ovarian insufficiency prior to age 40, is diagnosed in 20–30% of women with a PM (5). Fragile X-associated tremor-ataxia syndrome occurs in women and men with a PM, typically after age 60 (6, 7), although women have a lower absolute risk for FXTAS compared with men (8). Following the description of FXPOI in 1999 (9), medical comorbidities related to FXPOI's associated estrogen deficiency, such as osteoporosis and climacteric symptoms, were identified (5, 10). Additional studies have suggested that women who carry a PM are at higher risk for developing other health problems, including autoimmune disorders, chronic pain disorders, fibromyalgia, endocrine disorders, thyroid problems, hypertension, seizures, mental health disorders, and symptoms related to FXTAS (11–14). In addition to physical health conditions, mental health problems, such as anxiety and depression, have also been noted among PM carriers (15, 16). Hagerman and colleagues proposed the term FXAND to describe these varied mental health conditions (17). Movaghar et al. (18) also identified several mental health diagnoses in a review of 20,000 electronic health records, including agoraphobia, social phobia, and anxiety disorder, as occurring more frequently in PM women.

The cause of these varied health conditions is currently unknown. Increased comorbidity could be the result of the many challenges facing women who carry a PM, or it could be due to the biological impact of the PM itself. Previous studies have shown that caring for a child with IDD leads to higher levels of maternal stress relative to mothers of typically developing children due to the unique psychosocial, financial, and physical challenges (19–21). This elevated maternal stress can decrease maternal quality of life by elevating rates of depression and anxiety (20, 22–25). Also, many women with FXPOI struggle with infertility, which can affect quality of life and overall health (26, 27).

In our previous work, we characterized the comorbid conditions that were self-reported by 355 women with a PM (28). We identified 22 health conditions that were reported by at least 10% of women, with anxiety, depression, and headaches being the highest reported of these comorbid conditions (>30% of women). Further, we found that the number of conditions reported by women were significantly associated with two variables: body mass index (BMI) and a history of smoking. Cluster analysis was used to identify eight clusters of women who reported similar patterns of comorbid conditions.

The overall aim of the current work is to explore what predictor variables are associated with each of these 22 comorbid conditions. We have examined demographic variables, lifestyle and environmental variables, risk factors associated with carrying a PM allele, as well as the endorsement of depression and anxiety in each of these models. We collected self-report health and reproductive histories on 413 women with a PM. Based on our previous results (28), we hypothesized that BMI, smoking, depression, and anxiety would be associated with some of these comorbid conditions. Depression and anxiety were included in the analysis as predictor variables because we identified a stark contrast in the complexity of medical history for those who reported these mental health conditions in our previous cluster analysis compared to those that did not. This finding mirrors associations between mental health conditions and chronic conditions in the general population (29). However, this association is of particular interest in this population given the elevated rates of depression and anxiety in carriers of the PM allele (30). Interestingly, these predictor variables, as well as others such as age at interview, did show a significant association with a subset of the comorbid condition. The significant variables associated with each condition are presented.

## Methods

### Study Population

Emory University Institutional Review Board approved all protocols and consent forms, and informed consent was obtained from all participants. Participants were identified using different recruitment strategies: through previous Emory FXS research projects, recruitment at conferences for families with FXS, and through collaborations with other research groups who study FXS. Information was not systematically collected on which method of recruitment individuals came from. Once a family member was identified, additional family members were screened for eligibility without respect to phenotype. Eligibility was based on PM carrier status and sex. Biological samples were collected, and each participant completed a reproductive and health history questionnaire. These surveys were completed either through the mail or online. Data included general demographics (e.g., age at interview, race/ethnicity, education, income), lifestyle factors that might affect overall health (e.g., smoking, alcohol use, BMI), reproductive history (e.g., menstrual history, reason for cessation of menses, contraception use, pregnancy history), and general medical history. For the medical history, participants reported the presence or absence of various conditions by indicating 0: “I do not have this condition,” 1: “I think I have this but have not been diagnosed by a medical professional,” or 2: “I have been diagnosed with this by a medical professional.” If Option 2 was chosen, age at diagnosis was asked. Sixty-three conditions were queried on the medical history questionnaire. In our previous work, any condition reported by >10% of all women carrying a PM was included in further data analyses (28). For this analysis, a subset of conditions now fell below this frequency (Supplementary Table 1); however, we included them in the analysis for this paper.

The reproductive history was used to determine whether menses had stopped or if a woman was still having menstrual cycles. A dichotomous variable was created for FXPOI status: women with an age at natural menopause (AAM) < age 40 were defined as having FXPOI. Any women with iatrogenic (e.g., hysterectomy/oophorectomy) or alternative causes of menses cessation were not assigned a FXPOI status and were excluded from any model that included the FXPOI variable. Women were classified as having FXPOI if menses was absent for at least 4–6 months along with menopausal-level follicle-stimulating hormone (31). Women who were still having menstrual cycles or had menopause at age 40 or later were classified as not having FXPOI. For 103 women, a FXPOI assignment could not be made (e.g., women who had surgery, such as a hysterectomy, before age 40, women who were still cycling but younger than age 40, etc.) and were not included in the models below that included FXPOI.

### Laboratory Methods

Qiagen Qiamp DNA Blood Mini Kit, Gentra Puregene extraction kit, or prepIT-L2P protocol from Oragene were used to extract DNA from biological samples.

A fluorescent sequencer method was used to determine FRAXA CGG repeat numbers (32). A second PCR protocol was used for females with only one allele (33). The PCRs for FRAXA consisted of 1X PCR Buffer (Gibco/BRL), 10% dimethyl sulfoxide (DMSO), 370 μM deazaG, 500 μM d(ACT), 0.3 μM each primer, 15 ng T4 gene 32, and 1.05 U Roche Expand Long *Taq*. Primers for the FMR1 gene were C: 5′-GCTCAGCTCCGTTTCGGTTTCACTTCCGGT3-′, and F: 5′-AGCCCCGCACTTCCACCAGCTCCTCCA3-′ (34).

### Statistical Analysis

For the analyses, we combined Option 1 (“I think I have this but have not been diagnosed by a medical professional”) and 2 (“I have been diagnosed with this by a medical professional”) as a positive endorsement of each health condition to exclude the potential that an environmental factor or a mental health condition may impact the ability or willingness for a participant to seek a medical diagnosis. However, the information for the frequency of Option 1 vs. Option 2 is included in Supplementary Table 1, and models that only used Option 2 (“I have been diagnosed with this by a medical professional”) as a positive endorsement are shown in Supplementary Table 2.

#### Logistic Regression Analysis

For each comorbid condition, we tested a logistic regression model that tested up to 11 different predictor variables. First, we included four demographic variables: age at interview (continuous variable), race/ethnicity (binary variable for 1: White or 0: any other race/ethnicity or unknown), education (binary variable for 0: ≥some college completed compared to 1: no college), and income (binary variable for 1: <$50,000 annual income or 0: >$50,000). We also included three environmental/lifestyle variables: smoking (binary variable for having ever smoked = 1, never smoked = 0; or binary variable for currently smoking = 1, or not currently smoking = 0), alcohol use (binary variable for drinking more than one day a week = 1 or not = 0), and BMI (continuous variable). The third group of variables included PM-associated factors: *FMR1* CGG repeat size (continuous variable), a binary variable for whether or not they had a child with FXS, and a binary variable for a diagnosis of FXPOI. The final group of variables tested included endorsing depression or anxiety.

Of note, any final model that included FXPOI had a reduced sample size, because 103 women could not be assigned a FXPOI status. A backwards elimination strategy was used to define the final model with one distinction. Because the presence of FXPOI in the model impacted total sample size, we first tested the full model including FXPOI. If FXPOI was not significant in the full model, it was the first variable to be removed from the model. After this step, variables were removed from the model based on their *p*-value until all variables that remained in the model met the threshold of *p* < 0.05.

For all analyses of the reported conditions, a Bonferroni correction was used to assess significant differences. Because 22 total conditions were analyzed, a conservative *p*-value of 0.0023 was used as the threshold for significance; although we have included information for all predictor variables that met a threshold of *p* < 0.05 for descriptive purposes.

Linear regression models were used to test for associations of each of the predictor variables that were listed above with the total number of conditions reported. In this model, Anxiety and Depression were used as predictor variables, so they were not included in the sum variable for the total number of conditions reported. Similar to the logistic regression models, variables were eliminated using a backwards elimination strategy based on their association with the number of conditions. Only variables that were significant at *p* < 0.05 were included in the final model.

All regression models were also confirmed using generalized estimating equations (GEE) to adjust for relatedness of individuals within the dataset. *P*-values from the GEE models are included in Supplementary Tables 3, 4.

All analyses were done using SAS 9.4.

## Results

**Figure 1:**
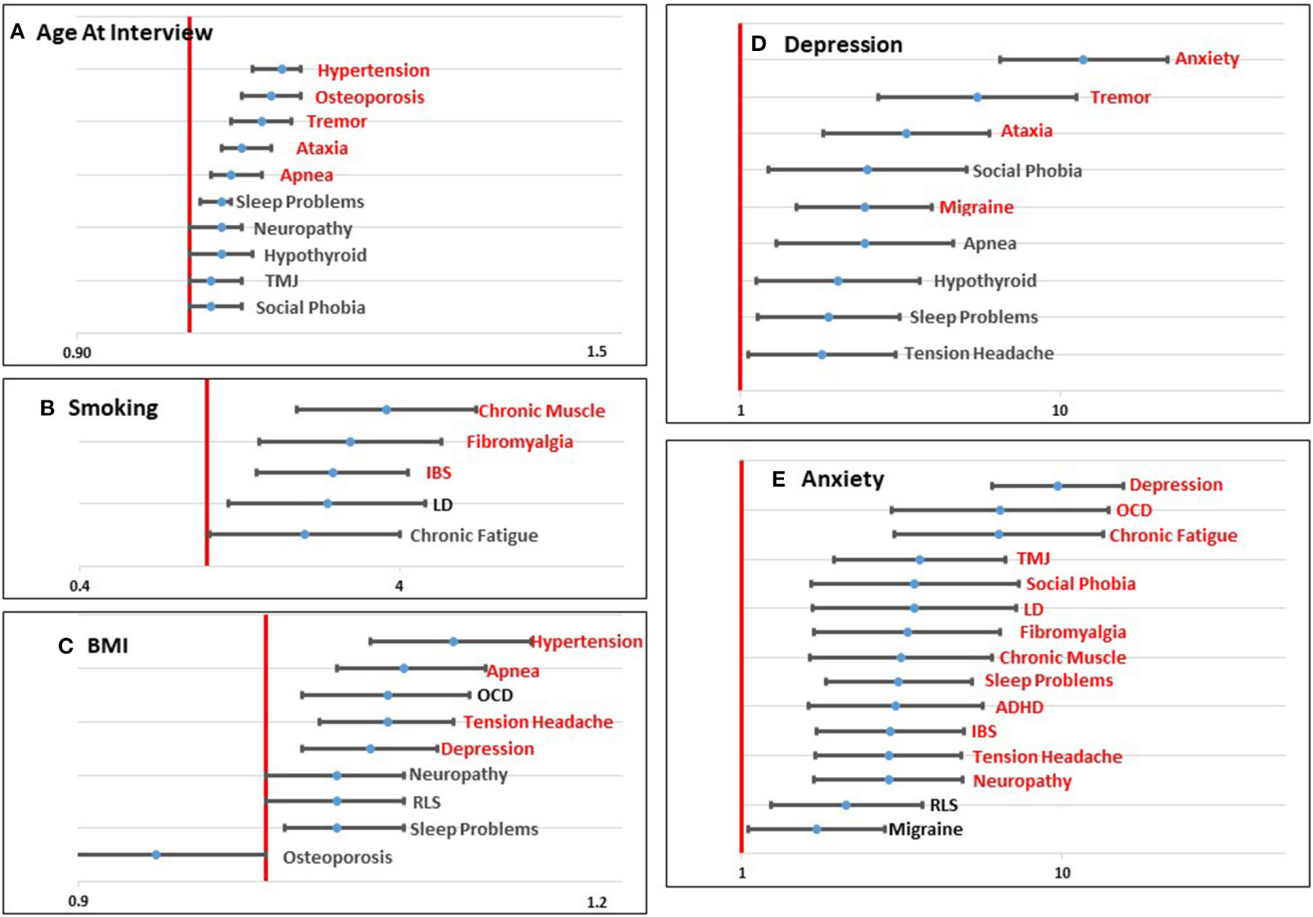
Graphical representation of significant (*p* < 0.0023; shown in red) and marginally significant (*p* < 0.05; shown in black) odds ratios for age at interview **(A)**, smoking **(B)**, BMI **(C)**, Depression **(D)**, and Anxiety **(E)** for each comorbid condition tested. Values to the left of the red line (1.0) indicate a decreased risk associated with the predictor variable and the comorbid condition. Values to the right of the red line indicate an increased risk associated with the predictor variable and the comorbid condition.

Basic descriptive information for our study population is shown in Table 1. In total, 413 women with a PM completed the reproductive and medical history questionnaire and provided a biological sample for *FMR1* genotyping. The reported frequencies of each of the comorbid conditions that were tested are shown in Table 2. For each comorbid condition, logistic regression was used to identify the variables that were associated with the endorsement of each of the comorbid conditions. In total, 22 models were tested, and Table 2 presents the odds ratios and 95% confidence intervals for the variables that were significant at our Bonferroni corrected *p*-value of 0.0023 (shown in red), as well as the variables that were marginally significant at *p* < 0.05 (shown in black). For variables that showed a significant association with at least three comorbid conditions, the odds ratios are presented graphically in Figure 1. All other variables except race/ethnicity, which was not significantly associated with any comorbid condition, are presented in Supplementary Figure 1. Below we discuss each category of predictor variables and the patterns that were seen.

**Table 1:** Self-reported demographic, lifestyle, and PM-associated variables.

**N**	**413**
**Age at Interview (Mean ± S.E.)**	48.1 ± 0.6 (19–93)
**Race (self-report)**	**%**
White	90.1
Black	3.5
Hispanic	0.5
Asian	3.2
Other/Not Stated/Unknown	2.7
**Education**	**%**
Some high school	1.0
High school degree/GED	6.5
Trade/Vocational school	4.1
Some college	15.2
College degree	39.5
Graduate or professional school degree	33.7
**Income**	**%**
<$10,000	1.2
$10,000–$25,000	3.9
$25,000–$50,000	15.1
$50,000–$75,000	21.7
$75,000–$100,000	12.9
>$100,000	38.8
Not stated/Unknown	6.4
**Ever Smoke (%)**	29.2
**Alcohol Use**	**%**
<1 day per week	64.0
1–2 days per week	16.5
3–4 days per week	11.6
5–6 days per week	5.1
7 days per week	2.8
**BMI (Mean ± S.E.)**	26.5 ± 0.3 (17.3–63.4)
**Repeat size (Mean ± S.E.)**	90.3 ± 0.8 (56–160)
**Has a child with FX (%)**	53.6
**FXPOI (%) (*****N*** **= 310)**	40.0

*PM, premutation; BMI, body mass index; FXPOI, Fragile X-associated primary ovarian insufficiency*.

**Table 2:** Prediction models for each comorbid condition.

		**Demographic variables**			**Environmental/Lifestyle variables**			**PM-associated risk factor variables**			**Mental health variables**	
	**% Positive endorsement**	**Age at interview**	**Education**	**Income**	**Smoking**	**Alcohol use**	**BMI**	**Repeat size**	**Has a FX child**	**FXPOI**	**Depression**	**Anxiety**
Anxiety	37.9	NS	NS	NS	NS	NS	NS	NS	2.21 (1.21–4.06)	3.03 (1.65–5.57)	11.83 (6.48–21.59)	
Depression	36.1	NS	NS	NS	NS	NS	1.06 (1.02–1.10)	NS	NS	NS		9.71 (6.03–15.62)
Migraine	34.3	NS	NS	NS	NS	NS	NS	NS	NS	NS	2.45 (1.50–3.98)	1.71 (1.05–2.78)
Tension Headache	30.7	NS	4.23 (1.71–10.46)	2.38 (1.33–4.24)	NS	NS	1.07 (1.03–1.11)	NS	NS	NS	1.80 (1.06–3.06)	2.87 (1.70–4.84)
Sleep Problems	28.4	1.03 (1.01–1.04)	NS	NS	NS	NS	NS	NS	NS	NS	1.89 (1.13–3.16)	3.09 (1.82–5.24)
Osteoporosis	20.7	1.08 (1.05–1.11)	NS	NS	NS	NS	0.94 (0.89–1.00)	NS	0.51 (0.28–0.93)	2.59 (1.38–4.87)	NS	NS
Neuropathy	19.9	1.02 (1.00–1.05)	NS	NS	NS	NS	1.05 (1.01–1.08)	1.03 (1.01–1.04)	NS	NS	NS	2.86 (1.67–4.90)
IBS	19.4	NS	NS	NS	2.47 (1.43–4.26)	0.46 (0.25–0.84)	NS	NS	NS	NS	NS	2.91 (1.71–4.95)
Hypothyroid	18.0	1.03 (1.00–1.06)	NS	NS	NS	NS	NS	NS	NS	2.13 (1.16–3.88)	2.01 (1.12–3.62)	NS
Hypertension	16.9	1.09 (1.06–1.11)	NS	NS	NS	NS	1.11 (1.06–1.16)	NS	NS	NS	NS	NS
RLS	15.7	NS	NS	NS	NS	NS	1.04 (1.00–1.08)	NS	NS	NS	NS	2.12 (1.23–3.66)
Ataxia	14.0	1.05 (1.03–1.08)	NS	NS	NS	NS	NS	1.02 (1.00–1.04)	NS	NS	3.29 (1.80–6.02)	NS
Apnea	12.6	1.04 (1.02–1.07)	NS	NS	NS	NS	1.08 (1.04–1.13)	NS	NS	NS	2.45 (1.30–4.61)	NS
TMJ	12.6	1.02 (1.00–1.05)	8.66 (1.16–64.77)	NS	NS	NS	NS	NS	NS	NS	NS	3.59 (1.93–6.67)
Social Phobia	12.3	1.02 (1.00–1.05)	NS	NS	NS	NS	NS	NS	NS	NS	2.49 (1.22–5.08)	3.46 (1.64–7.29)
Chronic Muscle	11.9	NS	NS	NS	3.64 (1.90–6.94)	0.38 (0.18–0.82)	NS	NS	NS	NS	NS	3.14 (1.63–6.03)
Fibromyalgia	11.6	NS	NS	2.04 (1.01–4.12)	2.80 (1.45–5.39)	0.45 (0.21–0.97)	NS	NS	NS	NS	NS	3.28 (1.68–6.39)
ADHD	11.4	NS	NS	NS	NS	NS	NS	NS	NS	NS	NS	3.02 (1.61–5.65)
Chronic Fatigue	10.9	NS	NS	NS	2.02 (1.02–4.01)	0.41 (0.18–0.92)	NS	NS	NS	NS	NS	6.36 (3.00–13.49)
Tremor	10.7	1.07 (1.04–1.10)	NS	NS	NS	NS	NS	NS	NS	NS	5.51 (2.70–11.25)	NS
OCD	9.7	NS	NS	NS	NS	NS	1.07 (1.02–1.12)	NS	NS	NS	NS	6.41 (2.94–14.00)
LD	9.0	NS	NS	2.39 (1.14–5.02)	2.37 (1.17–4.80)	NS	NS	NS	NS	NS	NS	3.46 (1.66–7.21)

*Stepwise logistic regression was used to determine which predictor variables were significant at p < 0.0023 (in red) or p < 0.05 (in black). All variables that were not significant (NS; p > 0.05) were not included in the final model. Odds ratios and 95% confidence intervals are shown for these predictor variables. PM, premutation; IBS, irritable bowel syndrome; RLS, restless leg syndrome; TMJ, temporomandibular joint dysfunction; ADHD, attention deficit-hyperactivity disorder; OCD, obsessive compulsive disorder; LD, learning disability. Race was also tested as a predictor variable but no significant associations were seen with any of the comorbid conditions*.

### Demographic Variables

Four demographic variables were included in the initial models: age at interview, race, education, and income. Not surprisingly, age at interview was significantly associated with comorbid conditions that have an increased risk with aging, including Hypertension, Osteoporosis, Tremor, Ataxia, and Sleep Apnea. Several other conditions showed a marginally significant association with age (Table 2; Figure 1A). Race was not significantly associated with any comorbid condition, likely due to the lack of racial diversity in our population with more than 88% of our population self-reporting as White. Lower level of education was significantly associated with increased risk of Tension Headaches and marginally associated with increased risk of Temporomandibular Joint Dysfunction (TMJ) (Table 2; Supplementary Figure 1A). Lower income was marginally associated with increased risk of Learning Disabilities (LD), Tension Headaches, and Fibromyalgia (Table 2; Supplementary Figure 1B).

### Environmental/Lifestyle Variables

Three environmental/lifestyle variables were also tested in each model: smoking, alcohol use, and BMI. Alcohol use did not meet the Bonferroni adjusted threshold for significance (Table 2; Supplementary Figure 1C), but was marginally significant for four conditions. In each case, drinking alcohol more than one day a week was marginally associated with lower risk for each of the comorbid outcomes. With respect to smoking, having ever smoked was significantly associated with an increased risk of Chronic Muscle pain, Fibromyalgia, and Irritable Bowel Syndrome (IBS), and it was marginally associated with LD and Chronic Fatigue (Table 2; Figure 1B). Higher BMI was significantly associated with increased risk of Hypertension, Apnea, Tension Headaches, and Depression. Marginal significant association of higher BMI was found for an increased risk of Obsessive Compulsive Disorder (OCD), Neuropathy, Restless Leg Syndrome (RLS), and Sleep Problems (Table 2; Figure 1C). For Osteoporosis, a marginally significant reduced risk was suggested with increased BMI.

### Risk Factors Associated With Carrying an FMR1 Premutation

We also tested three known risk factors that are associated with carrying a PM allele: *FMR1* repeat size, having a child with FXS, and FXPOI. Interestingly, increasing repeat size showed a significant association with risk of Neuropathy and a marginal association with Ataxia, two comorbid conditions that are seen in FXTAS patients (Table 2; Supplementary Figure 1D). Having a child with FXS showed a marginal association with an increased risk of Anxiety and a decreased risk of Osteoporosis (Table 2; Supplementary Figure 1E). Models that included FXPOI had a reduced sample size because 103 women did not have an assigned value for this variable (see Methods). Nevertheless, a diagnosis of FXPOI was significantly associated with an increased risk of Anxiety and marginally associated with Osteoporosis and Hypothyroidism (Table 2; Supplementary Figure 1F).

### Mental Health Variables

Based on our previous work (28), we found that self-reporting mental health conditions, such as Depression and Anxiety, was associated with more complex health profiles. Thus, we tested these variables as predictor variables for each of the other comorbid conditions. Depression was significantly associated with an increased risk of Anxiety, Tremor, Ataxia, and Migraine headaches, and marginally associated with Social Phobia, Sleep Apnea, Hypothyroidism, Sleep Problems, and Tension Headaches (Table 2; Figure 1D). Anxiety showed the highest number of associations of any of the variables with significant associations with 13 comorbid conditions and marginal associations with two additional comorbid conditions, all showing a positive association (Table 2; Figure 1E).

### Total Number of Comorbid Conditions

**Figure 2:**
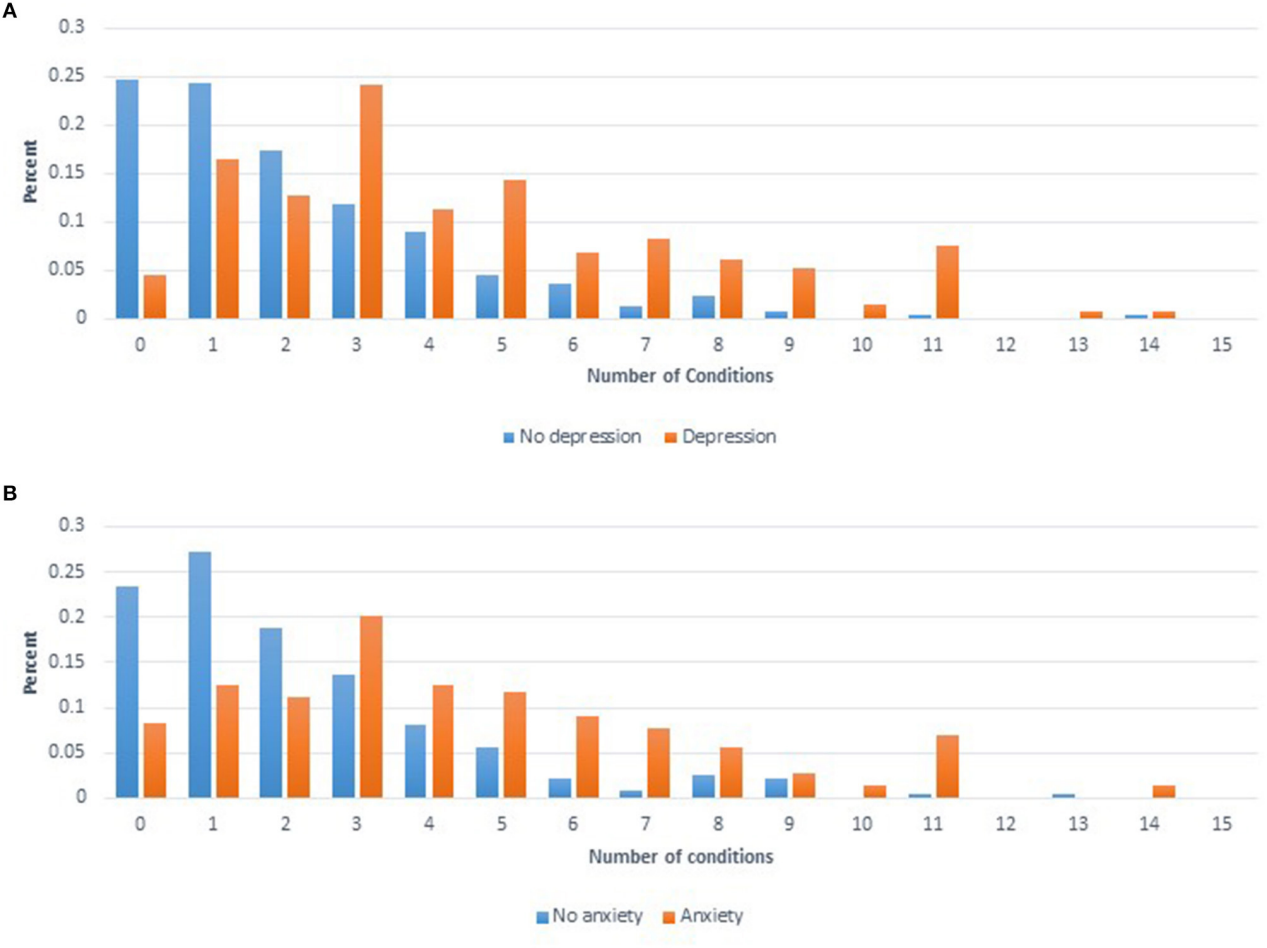
**(A)** Distribution of number of comorbid conditions reported for women who self-reported having depression (orange) compared to women that self-reported not having depression (blue). **(B)** Distribution of number of comorbid conditions reported for women who self-reported having anxiety (orange) compared to women that self-reported not having anxiety (blue).

To summarize the predictors of the overall health condition of each woman, the number of conditions reported per woman for 20 conditions were summed. Depression and Anxiety were excluded from this sum variable as they were used as predictor variables in the final model. The final model for the number of conditions included six significant variables: age at interview (*p* < 0.0001), income (*p* = 0.0019), smoking (*p* = 0.0035), BMI (*p* = 0.0007), Depression (*p* < 0.0001), and Anxiety (*p* < 0.0001). Figure 2 shows the distribution for the number of conditions reported by women divided by whether or not women self-reported Depression (Figure 2A) or Anxiety (Figure 2B).

## Discussion

In this work, we have presented findings about the predictor variables that are associated with each of the 22 comorbid conditions that we previously identified as occurring in ~10% or more of the women included in this study who carry a PM (28). We tested demographic variables, environmental/lifestyle variables, risk factors associated with carrying a PM, and mental health variables. By far, the most frequently associated variables with the various comorbid conditions were the mental health variables, Depression and Anxiety. Other variables that were found to be significantly associated with several comorbid conditions included age at interview, smoking, and BMI.

Age at interview was significantly associated with several comorbid conditions that have previously been associated with aging, including Hypertension, Osteoporosis, Tremor, Ataxia, and Apnea (35–39). An interesting pattern was observed for the comorbid conditions that were associated with smoking. Several of the conditions that are frequently reported by women with a PM that are associated with pain, including Chronic Muscle Pain, Fibromyalgia, and IBS, were significantly associated with smoking. Previous studies on all three of these conditions have also found an association with smoking and a higher prevalence of pain (40–43). This association could indicate a modifiable factor that could decrease the pain associated with these comorbid conditions in women with a PM. A variable for current smoking was also tested in each of these models as a follow up analysis; however, the variable for current smoking did not reach significance in any of these models. Importantly, in our dataset, we are unable to correlate the onset of comorbid conditions with the timing of smoking. BMI showed significant associations with Hypertension, Apnea, Tension Headaches, and Depression. Similar to our findings with age and smoking, many of these comorbid conditions have been linked to BMI in other studies, as well (44, 45).

Several of the other variables only showed significant or marginal associations with a few comorbid conditions. Of interest, repeat size was associated with two symptoms of FXTAS, Neuropathy and Ataxia, consistent with previous genotype/phenotype studies in FXTAS that show a positive linear relationship of repeat size with risk for FXTAS (46). In addition, as we presented in our previous work (28), FXPOI was significantly associated with Anxiety.

Anxiety and, to a lesser extent, Depression were significantly associated of many of the other comorbid conditions. Depression was associated with some of the conditions associated with FXTAS, such as Tremor and Ataxia. Anxiety was significantly associated with many of the highly reported conditions seen among PM carriers, including Chronic Fatigue, Fibromyalgia, and Chronic Muscle Pain. It is unclear from these results whether Depression or Anxiety are causative of these comorbid conditions or an effect of these complex health profiles. We only asked for the age of onset for these conditions for women that reported that they had received a diagnosis from a medical professional, and the mean age of onset for each of these comorbid conditions is included in Supplementary Table 1.

The results from these exploratory analyses indicate that there are several modifiable risk factors associated with comorbid conditions. Smoking and BMI are factors that can be addressed. As presented by Dr. Hagerman in her review of FXAND (17), selective serotonin reuptake inhibitors (SSRIs) or serotonin and norepinephrine reuptake inhibitors (SNRIs) can be used to treat anxiety and depression in PM carriers, and daily exercise can have a positive impact on mental health as well as BMI. By potentially addressing these mental health challenges with SSRIs, other comorbid conditions may also be ameliorated. Tassanakijpanich et al. recommend a multidisciplinary holistic approach to manage the health conditions associated with *FMR1* PM carriers (4).

The mechanism for these comorbid conditions is currently unknown. For FXTAS, current data support two non-mutually exclusive molecular pathogenesis mechanisms: transcribed PM alleles carry expanded CGG repeats that can be found in RNA foci (47) and/or inclusions (48), and the PM CGG repeat expansion induces RAN translation within the 5′ UTR of *FMR1* mRNA, producing polypeptides that may be toxic (49). In our data, repeat size of the PM was associated with Neuropathy and Ataxia (Table 2). Follow up molecular studies are the necessary next steps to identify factors that put particular women with a PM at risk.

There are several limitations to this research. Most notably, these data are based on self-report. The population that has participated in our research may have some biases: women with minimal health conditions may have more time and energy for participating in research, or conversely, women with more complicated health histories may have greater motivation to participate in research. Additionally, many of the families that participate in our research come to our attention at conferences, which potentially biases our sample towardz families of higher socioeconomic status. Further, the percentage of women with FXPOI in our dataset (40%) is higher than what would be expected, indicating that there may be some bias in women with FXPOI being more likely to contact us for participation in our research studies. Our study population is also not racially or ethnically diverse, an important factor given potential barriers to access to health care and subsequent receipt of a medical diagnosis across the population. We were also unable to adjust our models based on the method of recruitment (e.g., if individuals were recruited at a conference, through a family member, etc.) because we did not systematically track this information on all individuals. Our goal in this work was to understand the associated predictor variables for each of these comorbid conditions among women with a PM; however, similar data from women who do not carry a PM would help establish whether any of these variables have a differential impact on the background of having a PM allele. Also of note, our questionnaire was designed to ask about lifetime occurrence of these conditions. We were not able to distinguish the order of occurrence within women of each diagnosis to better understand what risk factors may be causative and which may be an effect of the specific comorbid condition. An additional limitation is that we have also combined the responses for women that think they have the comorbid condition with women who report being diagnosed by a medical professional. As an additional test of the data, we tested the same models for women who reported being diagnosed by a medical professional compared to all other women (Supplementary Table 2). In addition, we performed a comparison of the women diagnosed by a medical professional compared to only the women that reported they did not have the conditions (i.e., women who thought they had the condition but had not been diagnosed by a medical professional were excluded from the analysis), and all results were consistent with the results shown in Supplementary Table 2. For many of the models, the results were similar; although, several were limited by a reduced sample size for women that were considered positive for the comorbid condition.

There are also several positive attributes to the study design. First, all parts of the project could be completed through the mail or online, eliminating many barriers, such as socioeconomic barriers, childcare needs, travel, or barriers related to mental health problems that could reduce an individual's ability to interact directly with a study team. Also, this is not a clinic-based population and therefore not selected for existing health conditions for which women were seeking medical care.

In summary, we have followed up on our previous study of 22 comorbid conditions reported by women who carry an *FMR1* PM by performing exploratory analyses identifying the predictor variables that are specifically associated with each condition. The most commonly associated variables included age, smoking, BMI, and self-report of either depression or anxiety. Of note, more than half of the comorbid conditions studied were associated with women who self-reported having anxiety. Significantly, some of these risk factors are modifiable through lifestyle choices or medical intervention. Important next steps will be to conduct longitudinal studies or more comprehensive studies of medical records to provide information about the order and relationship of these comorbid conditions, as well as identifying if any of these possible interventions have a significant impact on the overall health of women with these complex medical profiles.

## Data Availability Statement

The original contributions presented in the study are included in the article/Supplementary Material, further inquiries can be directed to the corresponding author/s.

## Ethics Statement

The studies involving human participants were reviewed and approved by Emory University Institutional Review Board. The patients/participants provided their written informed consent to participate in this study.

## Author Contributions

EA, SS, JH, and KS were involved in the conceptualization of the project. Formal analyses and writing of the original draft was performed by EA. Data curation was performed by KC, LS, and HH. Laboratory analyses were performed by AA and WH. Writing and methodology were completed by EA, HH, JH, KS, and SS. Funding acquisition was done by EA and SS. All authors contributed to the article and approved the submitted version.

## Conflict of Interest

The authors declare that the research was conducted in the absence of any commercial or financial relationships that could be construed as a potential conflict of interest.

## Publisher's Note

All claims expressed in this article are solely those of the authors and do not necessarily represent those of their affiliated organizations, or those of the publisher, the editors and the reviewers. Any product that may be evaluated in this article, or claim that may be made by its manufacturer, is not guaranteed or endorsed by the publisher.

## Supplementary Material

Supplementary Figure 1 Graphical representation of significant (*p* < 0.0023; shown in red) and marginally significant (*p* < 0.05; shown in black) odds ratios for education **(A)**, income **(B)**, alcohol **(C)**, repeat size **(D)**, having a child with FXS **(E)**, and FXPOI **(F)**.

Supplementary Table 1 Reported frequencies of each comorbid condition.

Supplementary Table 2 Prediction models for each comorbid condition.

Supplementary Table 3 Prediction model *p*-values using GEE analysis to adjust for relatedness of individuals (see Methods) within the dataset for models presented in Table 2.

Supplementary Table 4 Prediction model *p*-values using GEE analysis to adjust for relatedness of individuals (see Methods) within the dataset for models presented in Supplementary Table 2.
