# Supplementary figures and images for: Predictors of Comorbid Conditions in Women Who Carry an FMR1 Premutation
Source: Front Psychiatry. 2021 Oct 1;12:715922. doi: 10.3389/fpsyt.2021.715922 (PMC8517131; doi:10.3389/fpsyt.2021.715922)

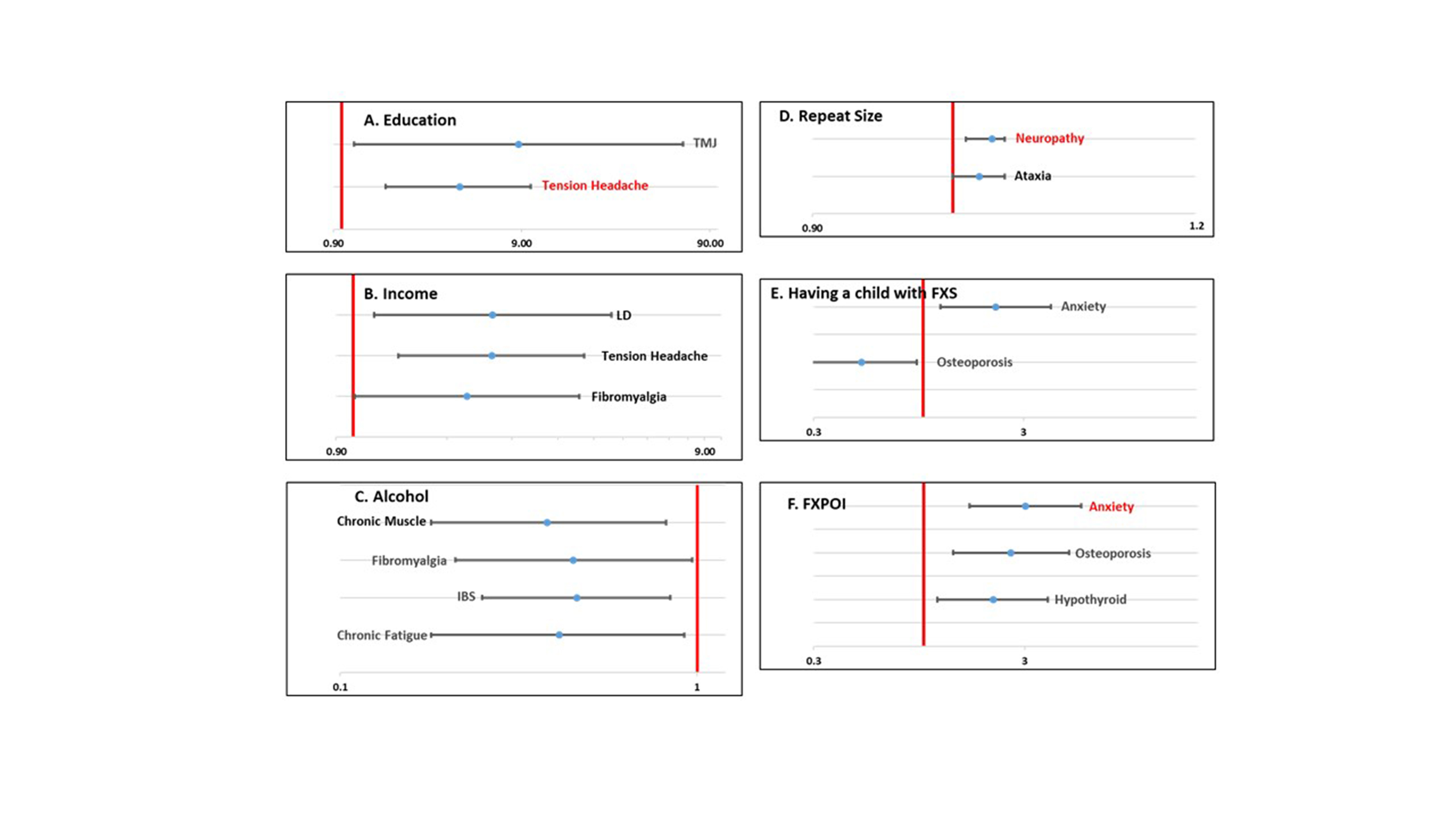

Supplement: Supplementary Figure 1 — Graphical representation of significant (p < 0.0023; shown in red) and marginally significant (p < 0.05; shown in black) odds ratios for education (A), income (B), alcohol (C), repeat size (D), having a child with FXS (E), and FXPOI (F). [file Image_1.jpg]
