# Supplementary material for: Predictors of Comorbid Conditions in Women Who Carry an FMR1 Premutation
Source: Front Psychiatry. 2021 Oct 1;12:715922. doi: 10.3389/fpsyt.2021.715922 (PMC8517131; doi:10.3389/fpsyt.2021.715922)
Supplement: Supplementary Table 1 — Reported frequencies of each comorbid condition. [file Data_Sheet_1.docx]

| Supplemental Table 1. Reported frequencies of each comorbid condition. | | | | | | |
| --- | --- | --- | --- | --- | --- | --- |
|  | Option 0^0^ | Option 1^1^ | Option 2^2^ | % Option 2 | % Option 1 and 2 | Mean ± SD for age of onset for Option 2^3^ |
| Anxiety | 256 | 62 | 94 | 22.8 | 37.9 | 33.4 ± 9.7 |
| Depression | 264 | 37 | 112 | 27.1 | 36.1 | 31.3 ± 11.8 |
| Migraine headaches | 270 | 51 | 90 | 21.9 | 34.3 | 24.7 ± 9.9 |
| Tension headaches | 285 | 89 | 37 | 9.0 | 30.7 | 27.7 ± 10.5 |
| Sleep problems | 295 | 83 | 34 | 8.2 | 28.4 | 38.0 ± 11.7 |
| Osteoporosis | 330 | 9 | 77 | 18.5 | 20.7 | 49.2 ± 11.4 |
| IBS | 333 | 32 | 48 | 11.6 | 19.4 | 32.0 ± 13.9 |
| Neuropathy | 330 | 58 | 24 | 5.8 | 19.9 | 51.4 ± 13.9 |
| Hypothyroid | 341 | 14 | 61 | 14.7 | 18.0 | 38.0 ± 14.1 |
| Hypertension | 343 | 2 | 68 | 16.5 | 16.9 | 44.7 ± 12.1 |
| Restless leg syndrome | 348 | 49 | 16 | 3.9 | 15.7 | 46.8 ± 14.9 |
| Ataxia | 355 | 41 | 17 | 4.1 | 14.0 | 53.2 ± 13.6 |
| Apnea | 361 | 22 | 30 | 7.3 | 12.6 | 47.3 ± 11.2 |
| TMJ | 361 | 9 | 43 | 10.4 | 12.6 | 29.9 ± 10.8 |
| Social phobia | 362 | 46 | 5 | 1.2 | 12.3 | 33.7 ± 4.7 |
| Fibromyalgia | 365 | 19 | 29 | 7.0 | 11.6 | 40.4 ± 11.0 |
| Chronic muscle pain | 363 | 30 | 19 | 4.6 | 11.9 | 40.0 ± 15.1 |
| ADHD | 366 | 32 | 15 | 3.6 | 11.4 | 28.1 ± 16.9 |
| Tremor | 369 | 31 | 13 | 3.1 | 10.6 | 55.7 ± 12.5 |
| Chronic fatigue syndrome | 368 | 37 | 8 | 1.9 | 10.9 | 29.7 ± 10.6 |
| OCD | 373 | 32 | 8 | 1.9 | 9.7 | 29.9 ± 7.2 |
| Learning disability | 375 | 28 | 9 | 2.2 | 9.0 | 13.6 ± 8.8 |
| Reynaud’s phenomenon | 379 | 13 | 20 | 4.8 | 8.0 | 31.1 ± 8.5 |
| Post-traumatic stress disorder | 383 | 14 | 16 | 3.9 | 7.3 | 38.1 ± 14.1 |
| Dysmenorrhea | 381 | 20 | 12 | 2.9 | 7.7 | 16.2 ± 3.8 |
| Limb movements in sleep | 386 | 24 | 2 | 0.5 | 6.3 | 46.5 ± 12.0 |
| Other phobia | 387 | 24 | 2 | 0.5 | 6.3 | 26.0 ± 22.6 |
| Mitral valve prolapse | 388 | 0 | 24 | 5.8 | 5.8 | 30.6 ± 15.4 |
| Rheumatoid arthritis | 387 | 11 | 14 | 3.4 | 6.1 | 43.1 ± 16.7 |
| Psoriasis | 390 | 8 | 15 | 3.6 | 5.6 | 34.0 ± 13.6 |
| Sensory Loss | 389 | 10 | 12 | 2.9 | 5.3 | 42.1 ± 15.2 |
| Autoimmune disease | 389 | 6 | 17 | 4.1 | 5.6 | 41.1 ± 13.4 |
| Urethral syndrome | 391 | 6 | 14 | 3.4 | 4.9 | 34.9 ± 14.4 |
| Other thyroid disorder | 394 | 1 | 17 | 4.1 | 4.4 | 37.6 ± 19.8 |
| Cancer | 401 | 0 | 15 | 3.6 | 3.6 | 50.0 ± 9.9 |
| Hyperthyroid | 398 | 5 | 11 | 2.7 | 3.9 | 33.9 ± 14.9 |
| Other neuromuscular disorder | 397 | 7 | 7 | 1.7 | 3.4 | 46.3 ± 8.8 |
| Type 2 diabetes | 402 | 1 | 13 | 3.1 | 3.4 | 51.5 ± 11.4 |
| Multiple chemical sensitivity | 399 | 12 | 2 | 0.5 | 3.4 | 25.0 ± 21.2 |
| Sjogren’s syndrome | 402 | 3 | 8 | 1.9 | 2.7 | 43.2 ± 10.1 |
| Myofascial pain syndrome | 401 | 7 | 4 | 1.0 | 2.7 | 41.7 ± 4.9 |
| Other endocrine disorder | 401 | 2 | 9 | 2.2 | 2.7 | 27.2 ± 9.4 |
| Seizures or epilepsy | 404 | 1 | 8 | 1.9 | 2.2 | 26.1 ± 14.2 |
| Alcoholism | 403 | 6 | 4 | 1.0 | 2.4 | 30.7 ± 7.1 |
| Brain Injury | 405 | 0 | 8 | 1.9 | 1.9 | 31.7 ± 17.8 |
| Stroke or TIA | 407 | 0 | 6 | 1.4 | 1.4 | 48.8 ± 12.0 |
| Pelvic Inflammatory Disease | 409 | 3 | 4 | 1.0 | 1.7 | 23.0 ± 1.6 |
| Optic Neuritis | 407 | 2 | 4 | 1.0 | 1.5 | 44.0 ± 15.1 |
| Lupus | 411 | 1 | 4 | 1.0 | 1.2 | 37.7 ± 10.1 |
| Multiple Sclerosis | 408 | 3 | 2 | 0.5 | 1.2 | 41.5 ± 12.0 |
| Meningitis or Encephalitis | 409 | 0 | 4 | 1.0 | 1.0 | 33.0 ± 20.4 |
| Brain Bleed | 409 | 1 | 3 | 0.7 | 1.0 | 38.0 ± 4.2 |
| Drug addiction | 409 | 1 | 2 | 0.5 | 0.7 | 25.0 ± 0 |
| Type I Diabetes | 414 | 0 | 2 | 0.5 | 0.5 | 19.0 ± 1.4 |
| Myotonic Dystrophy | 409 | 0 | 2 | 0.5 | 0.5 | 39.5 ± 3.5 |
| Oxygen Deprivation | 411 | 0 | 2 | 0.5 | 0.5 | 46.0 ± 65.0 |
| Parkinson’s Disease | 411 | 0 | 2 | 0.5 | 0.5 | 61.0 |
| Muscular Dystrophy | 411 | 0 | 1 | 0.2 | 0.2 | 52.0 |
| Autism spectrum disorder | 412 | 0 | 1 | 0.2 | 0.2 | 35.0 |
| Schizophrenia | 412 | 1 | 0 | 0 | 0.2 | N/A |
| Cerebral Palsy | 413 | 0 | 0 | 0 | 0 | N/A |
| Tourette’s or tic disorder | 413 | 0 | 0 | 0 | 0 | N/A |
| ^0^ number of subjects that selected “I do not have this condition”  ^1^ number of subjects that selected “I think I have this but have not been diagnosed by a medical professional”  ^2^ number of subjects that selected “I have been diagnosed with this by a medical professional”  ^3^ Age of onset was only asked for those that selected Option 2 “I have been diagnosed with this by a medical professional” | | | | | | |


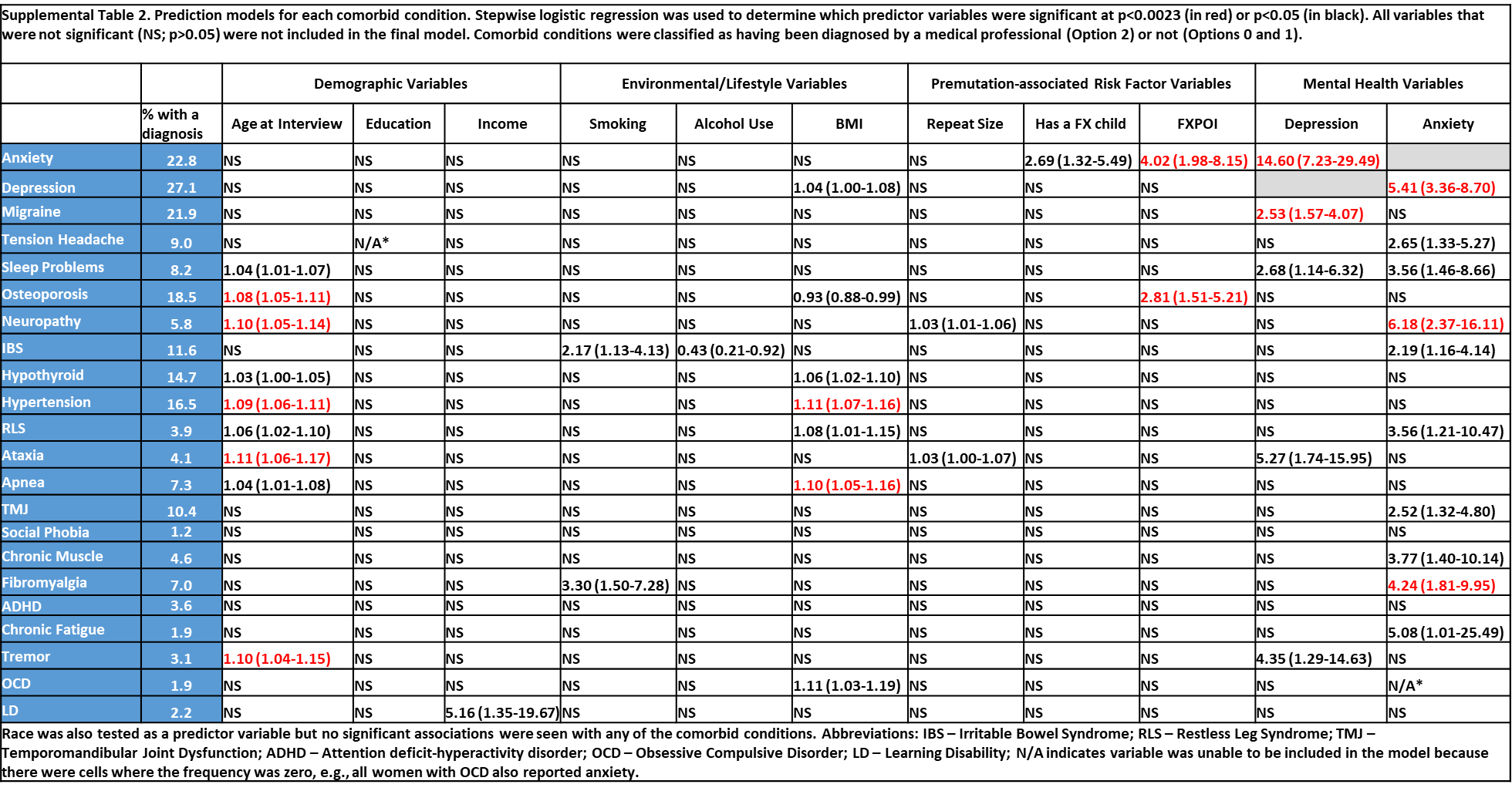


| **Supplemental Table 3. Prediction model p-values using GEE analysis to adjust for relatedness of individuals (see Methods) within the dataset for models presented in Table 2.** | | | | | | | | | | | | |
| --- | --- | --- | --- | --- | --- | --- | --- | --- | --- | --- | --- | --- |
|  | | **Demographic Variables** | | | **Environmental/Lifestyle Variables** | | | **PM-associated Risk Factor Variables** | | | **Mental Health Variables** | |
|  | **% Positive Endorsement** | **Age at Interview** | **Education** | **Income** | **Smoking** | **Alcohol Use** | **BMI** | **Repeat Size** | **Has a FX child** | **FXPOI** | **Depression** | **Anxiety** |
| **Anxiety** | **37.9** | **NS** | **NS** | **NS** | **NS** | **NS** | **NS** | **NS** | **0.0299** | **0.0015** | **<0.0001** |  |
| **Depression** | **36.1** | **NS** | **NS** | **NS** | **NS** | **NS** | **0.0001** | **NS** | **NS** | **NS** |  | **<0.0001** |
| **Migraine** | **34.3** | **NS** | **NS** | **NS** | **NS** | **NS** | **NS** | **NS** | **NS** | **NS** | **<0.0001** | **0.0080** |
| **Tension Headache** | **30.7** | **NS** | **0.0063** | **0.0374** | **NS** | **NS** | **0.0164** | **NS** | **NS** | **NS** | **NS** | **<0.0001** |
| **Sleep Problems** | **28.4** | **0.0029** | **NS** | **NS** | **NS** | **NS** | **NS** | **NS** | **NS** | **NS** | **0.0026** | **<0.0001** |
| **Osteoporosis** | **20.7** | **<0.0001** | **NS** | **NS** | **NS** | **NS** | **0.0227** | **NS** | **0.0310** | **0.0024** | **NS** | **NS** |
| **Neuropathy** | **19.9** | **0.0136** | **NS** | **NS** | **NS** | **NS** | **0.0246** | **0.0095** | **NS** | **NS** | **NS** | **<0.0001** |
| **IBS** | **19.4** | **NS** | **NS** | **NS** | **0.0001** | **0.0235** | **NS** | **NS** | **NS** | **NS** | **NS** | **<0.0001** |
| **Hypothyroid** | **18.0** | **0.0303** | **NS** | **NS** | **NS** | **NS** | **NS** | **NS** | **NS** | **0.0147** | **0.0325** | **NS** |
| **Hypertension** | **16.9** | **<0.0001** | **NS** | **NS** | **NS** | **NS** | **0.0003** | **NS** | **NS** | **NS** | **NS** | **NS** |
| **RLS** | **15.7** | **NS** | **NS** | **NS** | **NS** | **NS** | **0.0162** | **NS** | **NS** | **NS** | **NS** | **0.0020** |
| **Ataxia** | **14.0** | **<0.0001** | **NS** | **NS** | **NS** | **NS** | **NS** | **0.0012** | **NS** | **NS** | **<0.0001** | **NS** |
| **Apnea** | **12.6** | **<0.0001** | **NS** | **NS** | **NS** | **NS** | **0.0017** | **NS** | **NS** | **NS** | **0.0015** | **NS** |
| **TMJ** | **12.6** | **0.0159** | **0.0378** | **NS** | **NS** | **NS** | **NS** | **NS** | **NS** | **NS** | **NS** | **<0.0001** |
| **Social Phobia** | **12.3** | **0.0144** | **NS** | **NS** | **NS** | **NS** | **NS** | **NS** | **NS** | **NS** | **0.0010** | **0.0002** |
| **Chronic Muscle** | **11.9** | **NS** | **NS** | **NS** | **0.0007** | **0.0070** | **NS** | **NS** | **NS** | **NS** | **NS** | **0.0001** |
| **Fibromyalgia** | **11.6** | **NS** | **NS** | **0.0067** | **0.0009** | **NS** | **NS** | **NS** | **NS** | **NS** | **NS** | **<0.0001** |
| **ADHD** | **11.4** | **NS** | **NS** | **NS** | **NS** | **NS** | **NS** | **NS** | **NS** | **NS** | **NS** | **<0.0001** |
| **Chronic Fatigue** | **10.9** | **NS** | **NS** | **NS** | **NS** | **NS** | **NS** | **NS** | **NS** | **NS** | **NS** | **<0.0001** |
| **Tremor** | **10.7** | **<0.0001** | **NS** | **NS** | **NS** | **NS** | **NS** | **NS** | **NS** | **NS** | **<0.0001** | **NS** |
| **OCD** | **9.7** | **NS** | **NS** | **NS** | **NS** | **NS** | **0.0088** | **NS** | **NS** | **NS** | **NS** | **<0.0001** |
| **LD** | **9.0** | **NS** | **NS** | **0.0221** | **NS** | **NS** | **NS** | **NS** | **NS** | **NS** | **NS** | **<0.0001** |
| **Race was also tested as a predictor variable but no significant associations were seen with any of the comorbid conditions. Abbreviations: PM – Premutation; IBS – Irritable Bowel Syndrome; RLS – Restless Leg Syndrome; TMJ – Temporomandibular Joint Dysfunction; ADHD – Attention deficit-hyperactivity disorder; OCD – Obsessive Compulsive Disorder; LD – Learning Disability** | | | | | | | | | | | | |

| **Supplemental Table 4. Prediction model p-values using GEE analysis to adjust for relatedness of individuals (see Methods) within the dataset for models presented in Supplemental Table 2.** | | | | | | | | | | | | |
| --- | --- | --- | --- | --- | --- | --- | --- | --- | --- | --- | --- | --- |
|  | | **Demographic Variables** | | | **Environmental/Lifestyle Variables** | | | **PM-associated Risk Factor Variables** | | | **Mental Health Variables** | |
|  | **% with a diagnosis** | **Age at Interview** | **Education** | **Income** | **Smoking** | **Alcohol Use** | **BMI** | **Repeat Size** | **Has a FX child** | **FXPOI** | **Depression** | **Anxiety** |
| **Anxiety** | **22.8** | **NS** | **NS** | **NS** | **NS** | **NS** | **NS** | **NS** | **0.0145** | **0.0005** | **<0.0001** |  |
| **Depression** | **27.1** | **NS** | **NS** | **NS** | **NS** | **NS** | **0.0036** | **NS** | **NS** | **NS** |  | **<0.0001** |
| **Migraine** | **21.9** | **NS** | **NS** | **NS** | **NS** | **NS** | **NS** | **NS** | **NS** | **NS** | **<0.0001** | **NS** |
| **Tension Headache** | **9.0** | **NS** | **NS** | **NS** | **NS** | **NS** | **NS** | **NS** | **NS** | **NS** | **NS** | **0.0049** |
| **Sleep Problems** | **8.2** | **<0.0001** | **NS** | **NS** | **NS** | **NS** | **NS** | **NS** | **NS** | **NS** | **0.0181** | **0.0079** |
| **Osteoporosis** | **18.5** | **<0.0001** | **NS** | **NS** | **NS** | **NS** | **0.0388** | **NS** | **NS** | **0.0012** | **NS** | **NS** |
| **Neuropathy** | **5.8** | **<0.0001** | **NS** | **NS** | **NS** | **NS** | **NS** | **NS** | **NS** | **NS** | **NS** | **0.0005** |
| **IBS** | **11.6** | **NS** | **NS** | **NS** | **0.0089** | **0.0491** | **NS** | **NS** | **NS** | **NS** | **NS** | **0.0074** |
| **Hypothyroid** | **14.7** | **0.0496** | **NS** | **NS** | **NS** | **NS** | **NS** | **NS** | **NS** | **0.0240** | **NS** | **NS** |
| **Hypertension** | **16.5** | **<0.0001** | **NS** | **NS** | **NS** | **NS** | **0.0003** | **NS** | **NS** | **NS** | **NS** | **NS** |
| **RLS** | **3.9** | **0.0011** | **NS** | **NS** | **NS** | **NS** | **0.0361** | **NS** | **NS** | **NS** | **NS** | **0.0341** |
| **Ataxia** | **4.1** | **<0.0001** | **NS** | **NS** | **NS** | **NS** | **NS** | **NS** | **NS** | **NS** | **0.0018** | **NS** |
| **Apnea** | **7.3** | **<0.0001** | **NS** | **NS** | **NS** | **NS** | **0.0004** | **NS** | **NS** | **NS** | **NS** | **NS** |
| **TMJ** | **10.4** | **NS** | **NS** | **NS** | **NS** | **NS** | **NS** | **NS** | **NS** | **NS** | **NS** | **0.0028** |
| **Social Phobia** | **1.2** | **NS** | **NS** | **NS** | **NS** | **NS** | **NS** | **NS** | **NS** | **NS** | **NS** | **NS** |
| **Chronic Muscle** | **4.6** | **NS** | **NS** | **NS** | **NS** | **NS** | **NS** | **NS** | **NS** | **NS** | **NS** | **0.0002** |
| **Fibromyalgia** | **7.0** | **NS** | **NS** | **NS** | **0.0103** | **NS** | **NS** | **NS** | **NS** | **NS** | **NS** | **0.0010** |
| **ADHD** | **3.6** | **NS** | **NS** | **NS** | **NS** | **NS** | **NS** | **NS** | **NS** | **NS** | **NS** | **NS** |
| **Chronic Fatigue** | **1.9** | **NS** | **NS** | **NS** | **NS** | **NS** | **NS** | **NS** | **NS** | **NS** | **NS** | **0.0516** |
| **Tremor** | **3.1** | **<0.0001** | **NS** | **NS** | **NS** | **NS** | **NS** | **NS** | **NS** | **NS** | **0.0038** | **NS** |
| **OCD** | **1.9** | **NS** | **NS** | **NS** | **NS** | **NS** | **0.0024** | **NS** | **NS** | **NS** | **NS** | **N/A*** |
| **LD** | **2.2** | **NS** | **NS** | **0.0027** | **NS** | **NS** | **NS** | **NS** | **NS** | **NS** | **NS** | **NS** |
| **Race was also tested as a predictor variable but no significant associations were seen with any of the comorbid conditions. Abbreviations: PM – Premutation; IBS – Irritable Bowel Syndrome; RLS – Restless Leg Syndrome; TMJ – Temporomandibular Joint Dysfunction; ADHD – Attention deficit-hyperactivity disorder; OCD – Obsessive Compulsive Disorder; LD – Learning Disability; N/A indicates variable was unable to be included in the model because there were cells where the frequency was zero, e.g., all women with OCD also reported anxiety.** | | | | | | | | | | | | |
